# Assessing adaptive and plastic responses in growth and functional traits in a 10‐year‐old common garden experiment with pedunculate oak (*Quercus robur* L.) suggests that directional selection can drive climatic adaptation

**DOI:** 10.1111/eva.13034

**Published:** 2020-06-18

**Authors:** Jan‐Peter George, Guillaume Theroux‐Rancourt, Kanin Rungwattana, Susanne Scheffknecht, Nevena Momirovic, Lea Neuhauser, Lambert Weißenbacher, Andrea Watzinger, Peter Hietz

**Affiliations:** ^1^ Department of Forest Genetics Federal Research and Training Centre for Forests Natural Hazards and Landscape (BFW) Vienna Austria; ^2^ Institute of Botany University of Applied Life Sciences and Natural Resources Vienna (BOKU) Vienna Austria; ^3^ Institute of Soil Research University of Applied Life Sciences and Natural Resources Vienna (BOKU) Vienna Austria; ^4^ Present address: Tartu Observatory University of Tartu Tõravere Estonia; ^5^ Present address: Department of Botany Faculty of Science Kasetsart University Bangkok Thailand

**Keywords:** adaptive plasticity, functional traits, genotype‐by‐environment interactions, heritability, local adaptation, tree growth

## Abstract

Understanding how tree species will respond to a future climate requires reliable and quantitative estimates of intra‐specific variation under current climate conditions. We studied three 10‐year‐old common garden experiments established across a rainfall and drought gradient planted with nearly 10,000 pedunculate oak (*Quercus robur* L.) trees from ten provenances with known family structure. We aimed at disentangling adaptive and plastic responses for growth (height and diameter at breast height) as well as for leaf and wood functional traits related to adaptation to dry environments. We used restricted maximum likelihood approaches to assess additive genetic variation expressed as narrow‐sense heritability (h^2^), quantitative trait differentiation among provenances (Q_ST_), and genotype‐by‐environment interactions (GxE). We found strong and significant patterns of local adaptation in growth in all three common gardens, suggesting that transfer of seed material should not exceed a climatic distance of approximately 1°C under current climatic conditions, while transfer along precipitation gradients seems to be less stringent. Moreover, heritability reached 0.64 for tree height and 0.67 for dbh at the dry margin of the testing spectrum, suggesting significant additive genetic variation of potential use for future selection and tree breeding. GxE interactions in growth were significant and explained less phenotypic variation than origin of seed source (4% versus 10%). Functional trait variation among provenances was partly related to drought regimes at provenances origins but had moderate explanatory power for growth. We conclude that directional selection, either naturally or through breeding, is the most likely and feasible outcome for pedunculate oak to adapt to warmer and drier climate conditions in the future.

## 1 INTRODUCTION

Intra‐specific trait variation (ITV) is an important feature in evolutionary biology as it is the result of several evolutionary forces that have worked on phenotypic variation in the past and provides the raw material for ongoing adaptation of species to various selective forces (Alberto et al., 2013; Benito Garzón, Alía, Robson, & Zavala, 2011; Bolnick et al., 2011). ITV comprises several sources of evolutionary drivers, including long‐term selection, historic gene flow, and random genetic drift, which have left their particular imprints in phenotypes and genotypes (Albert, Grassein, Schurr, Vieilledent, & Violle, 2011). Moreover, given that individuals and populations are also characterized by the ability to change their phenotype depending on the environment they are exposed to, plastic responses and, in particular, genetic variation in plasticity (GxE) constitutes another important source of ITV. The latter is of notable importance for sessile organisms such as trees, since their natural migration velocity is certainly too slow to track their ecological optimum when environmental conditions change rapidly as expected under climate change (Aitken, Yeaman, Holliday, Wang, & Curtis‐McLane, 2008; Bussotti, Pollastrini, Holland, & Brueggemann, 2015; Ghalambor, McKay, Carroll, & Reznick, 2007; Nicotra et al., 2010; Via & Lande, 1985). Disentangling adaptive and plastic responses in trees is of particular importance for climate adaptation and adaptive forest management, as well as for defining conservation goals for rear‐edge tree populations (i.e., populations at the trailing edge of a distribution) under climate change, because both will have different implications for future ecosystem management (e.g., Aitken & Bemmels, 2016; Fady et al., 2016). The presence of adaptive variation can mean that trait variation is heritable and can therefore be passed on from one generation to the next, but also that populations probably experienced spatially varying selection in the past and therefore show divergence in their mean trait values in space. High heritability may suggest that breeding programs for more resilient genotypes are desirable (Harfouche et al., 2012), whereas strong quantitative trait differentiation among populations (e.g., Q_ST_) implies that climatically preadapted genotypes exist and may be utilized in assisted gene flow and assisted migration schemes (Aitken & Bemmels, 2016).

Different approaches have been used to investigate adaptive or plastic responses in plants such as studying trait variation across landscapes (e.g., Porth et al., 2015) and establishing common garden experiments, where ecotypes or provenances of the same species grow under equal environmental conditions (e.g., Sáenz‐Romero et al., 2017). When replicated across several contrasting environments, common garden experiments can assess adaptive and plastic responses at the same time, assuming that a known family structure exists among trees within provenances (Matesanz & Valladares, 2014). Here, we analyzed data from three common garden experiments in which nearly 10,000 trees with known pedigree and provenance were planted across a rainfall gradient. Trees were analyzed for growth (height and diameter at breast height 10 years after planting) as well as for a number of functional traits with known importance for drought adaptation to assess the relative contributions of the various evolutionary drivers outlined above. We studied pedunculate oak (*Quercus robur* L.), a widespread wind‐pollinated temperate forest tree in Europe that can reach ages of up to 800 years and that has considerable importance for the forest industry as well as for forest ecosystem functions in Europe (Ducousso & Bordacs, 2004). Pedunculate oak is a largely outcrossing tree species that has survived the last glacial maximum within three big refugia in the Balkan peninsula, southern Italy, and Iberia (Petit et al., 2002) and occurs largely sympatric with its closely related congener sessile oak (*Quercus petraea)* resulting in contact zones where inter‐specific gene flow is realized (Petit, Bodénès, Ducousso, Roussel, & Kremer, 2004). Recent studies showed that *Q. petraea* exhibits significant imprints of local adaptation across the range of its distribution, that is, highest fitness was achieved where the variation between growth and provenance climate was small, and that the climate at seed origin explains a significant part of the phenotypic variation (Sáenz‐Romero et al., 2017). Here, we test whether such a pattern holds true for its closely related congener on a smaller geographic scale by integrating functional traits with known importance for drought adaptation. Additionally, our study goes beyond the provenance level and takes into account putative additive variance and plasticity attributable to the effects of families (i.e., mother trees). This permits to disentangle three sources of variation, that is, provenance‐adaptive, single‐tree‐adaptive, and GxE, all of which have different implications for future management in a changing climate. For example, current national seed transfer guidelines of forest reproductive material in Europe still recommend the use of local seed sources following a *“local is best”* paradigm (e.g., Konnert et al., 2015), even though seed sources from warmer and probably drier regions might help to mitigate consequences of ongoing warming and progressively drier vegetation periods in the near future.

We hypothesize that populations of pedunculate oak exhibit adaptation to the local climate so that growth would decrease from the local maximum with increasing climatic distance from the seed source (Savolainen, Pyhäjärvi, & Knürr, 2007). Additionally, we hypothesize that heritability in growth traits is significant within and across provenances of pedunculate oak and can be potentially utilized in tree breeding. Finally, we tested whether functional traits in leaves and wood that are known to be involved in drought adaptation can be used to explain growth differences among or within populations and may be used as candidates for selecting more resilient trees in the wild or in large‐scale progeny tests.

## 2 MATERIALS AND METHODS

### 2.1 Plant material

The trees studied are part of a national provenance test series, in which several provenances of pedunculate oak are tested across five common garden experiments. For the current study, a subset of three common gardens and ten provenances were selected to provide a suitable bioclimatic gradient for both provenances and testing sites (Table 1). Briefly, the three test sites (Wels, Weyerburg, and Weistrach) belong to three different ecozones according to the Austrian forest seed zone classification (Kilian, Müller, & Starlinger, 1994) and follow an annual rainfall and continentality gradient from 590 mm (Weyerburg, hereafter called the “dry site”) to 770 mm (Wels, “moist site”), with summer drought periods increasing from moist to dry sites. Seeds from 22 mother trees were collected in each of the ten provenances and sown in 2006 in an experimental nursery in Vienna. Mother trees were collected from registered local seed stands in Austria, Slovenia, Croatia, and Czech Republic. Plants were brought to the testing sites as 1‐year‐old seedlings in planting containers and were planted in a 2 × 1 m distance matrix with a total of 110 plants in each provenance cell. Each provenance was replicated three times in each common garden with each of the 22 mother trees being randomly represented five times in each cell. To account for family‐level variation, a mother tree identifier matrix was created for identifying families in each of the cells. In total, 9,900 trees were planted and grown over the observation period of 10 years in the three common gardens (Figure S1). Testing sites were regularly visited in the first years to remove competing vegetation (e.g., grasses and blackberry) in order to keep the seedling survival rate homogenous among sites, but no thinning or any other silvicultural treatment was applied in the first ten years.

**TABLE 1:** Overview of analyzed provenances and trial sites

Prov	Climatic Cluster	Name	Region	Lat	Lon	MAT	MAP	MWMT	MCMT
1	2	Geinberg	Austria‐north	48.28	13.31	8.69	890.66	19.05	−2.64
2	2	Linz	Austria‐north	48.33	14.29	8.90	815.02	19.50	−2.78
8	2	Rainfeld	Austria‐north	48.04	15.73	8.79	762.58	19.27	−2.55
6	1	Braunsberger Wald	Austria‐northeast	48.47	16.33	9.36	627.99	20.17	−2.69
12	1	Luising	Austria‐southeast	47.02	16.48	9.58	614.18	20.49	−2.46
14	4	Klagenfurt	Austria‐south	46.63	14.35	8.35	1,009.52	19.46	−4.24
17	5	Hluboka (CZ)	Czech Republic	49.09	14.44	7.36	497.90	17.92	−4.10
18	3	Kutina (HR)	Croatia‐west	45.43	16.68	10.85	837.72	21.64	−1.42
21	3	Velika Gorica (HR)	Croatia‐west	45.67	16.16	10.69	920.34	21.54	−1.59
19	3	Murska suma (SLO)	Slovenia‐north	46.50	16.51	9.83	802.68	20.74	−2.41

Site	Climatic Cluster	Category	Region	Lat	Lon	MAT	MAP	PDM	SHM
WB	1	Dry	Austria‐northeast	48.56	16.17	9.118329	597.93	69.83	59.20165634
WS	2	Intermediate	Austria central	48.05	14.56	8.963233	655.08	82.73	42.62147141
WL	2	Moist	Austria‐northwest	48.19	13.99	8.837837	773.87	89.36	37.35188293

Abbreviation: MAT, mean annual temp.; MAP, mean annual prec.; MWMT, mean warmest month temp.; MCMT, mean coldest month temp.; *N*, number of trees planted/analyzed; PDM: Prec. of driest month; SHM, summer heat:moisture index.

### 2.2 Climate data

Provenances and testing sites were climatically characterized by using long‐term climate variables that were derived from a 10 × 10 km downscaled EUROCORDEX climate dataset (Jacob et al., 2014). Briefly, climate data were spatially downscaled to a 1km^2^ resolution by applying the method described in Hamann, Wang, Spittlehouse, and Murdock (2013) and which is available in the ClimateEU database (available at http://tinyurl.com/ClimateEU *)*. The downscaled climate data were validated with observation data from the E‐obs dataset (Klok & Tank, 2009) with a correlation coefficient of 0.93 (Chakraborty, 2019). We used a subset of 13 climate variables (Table S1) to assign provenances to climatic clusters with similar long‐term growing conditions. For this, the elbow criterion for selecting the most likely number of clusters by visually inspecting the screeplot after performing a principal component analysis was applied. Analysis was carried out in R (R Development Core Team, 2008), and functions *prcomp* and *autoplot* from the *cluster* package were used for visualization (Maechler, Rousseeuw, Struyf, Hubert, & Hornik, 2019).

### 2.3 Traits

Diameter at breast height (dbh) and height after 10 years were measured in winter 2017/18 for all living trees by using a diameter measuring tape and a Vertex hypsometer (Haglof, Sweden), respectively. We additionally assessed leaf and wood functional traits that have been found to be involved in drought adaptation (see O'Brien et al., 2017; Ramírez‐Valiente, Etterson, Deacon, & Cavender‐Bares, 2018) for a subset of trees. Since functional traits are more time‐consuming to measure than tree size, we measured only nine unrelated individuals per provenance per site to capture trait variation within and among provenances, but without accounting for family structure within sites. The nine trees per provenance were always evenly sampled across the three blocks with three different families sampled in each block. However, across the three sites, half‐siblings from the same nine mother trees per provenance were sampled so that each family was represented by three trees. This resulted in functional trait data for 270 trees in total.

One wood sample per tree was collected at c. 1 m height with 5.15‐mm increment borers in February 2018. Leaves from the same trees were collected after having reached full expansion, on June 18–19, 2018, from one sun exposed branch per tree. To avoid water loss during transport, wood samples were transported in 2.5 ml plastic vials and leaves in wet plastic bags.

For wood samples, 30‐μm transverse sections were first cut with a rotary microtome (Leica Biosystems, United States), stained (safranin/ astrablue), and embedded in Euparal (Carl Roth, Germany). Digital images with pixel size of 1.157 µm were taken with a DM5500B transmission light microscope (Leica, Germany). Vessels of the outer three growth rings (representing years 2015–2017) were marked manually in Adobe Photoshop CS6 (Adobe Systems, USA) and measured automatically with ImageJ (Schneider, Rasband, & Eliceiri, 2012). We calculated mean vessel area (VA), the 95% quantile of vessel area (VA_max_) to represent the much larger earlywood vessels of oak, vessel lumen fraction (F, the sum of vessel lumina per cross‐sectional area), and theoretical hydraulic conductivity (Kh), calculated based on the Hagen–Poiseuille law (Tyree & Zimmermann, 2013) as Kh = (πρ_w_/128 η) × VD × D_h_^4^, where ρ_w_ is the density of water (998.2 kg/m^3^ at 20°), η the viscosity of water (1.002 × 10^−3^ Pa s at 20°C), VD vessel density (vessels m^−2^), and D_h_ = (Σ D^4^/n)/4, where D is the average of minor and major axes of the diameter of each individual vessels and n the number of measured vessels.

Ten large leaves per branch were rehydrated for 24 h between wet paper towels in a dark room at 4°C. After removing the petioles, leaves were carefully blotted dry, saturation weight was measured with an electronic balance to 0.1 mg, and leaves were scanned with a desktop scanner at 150 dpi resolution and dried for 72 hr at 60°C. Specific leaf area (SLA, g/cm^2^) was calculated as leaf dry mass/leaf area and leaf dry matter content (LDMC, g/g) as dry weight/ saturation weight. Subsamples of all ten leaves per branch (9 mm disk) were collected for stable carbon isotope analysis, a proxy for photosynthetic water‐use efficiency integrated over the growth and expansion of the leaf (Farquhar, Ehleringer, & Hubick, 1989). Subsamples were pooled, ground to a fine powder in a ball mill (TissueLyser 2, Qiagen, USA) and analyzed in an isotope ratio mass spectrometer (Delta V Advantage; Thermo Scientific, USA). The 13C:12C ratio and C content of the plant samples were measured by elemental analyser–isotopic ratio mass spectrometer (EA‐IRMS) with a FlashEA 1,112 connected to an IRMS Delta V advantage via a Conflow IV (Thermo Fisher Scientific, Bremen, Germany). C content was calibrated using a certified acetanilide standard. Stable isotope referencing was done with working standards referenced against the international certified standards NBS 22, IAEA‐CH‐6, IAEA‐600, IAEA‐NO‐3, and IAEA‐N‐1. The isotopic composition of C is reported in delta (δ) notation relative to the Vienna Pee Dee Belemnite (VPDB). The precision of the measurements is ≤0.2%.

One additional leaf per sampled tree was rehydrated for stomatal and vein density measurements. Stomatal imprints were made by pressing the fresh leaf on a small acrylic plate the size of a microscope slide covered by a drop of butanone. Both abaxial and adaxial sides of the leaf were pressed onto the slide, but no stomata were observed on the adaxial side. At least 5 images were collected per imprint (image size: 0.266 mm^2^; pixel size: 0.291 µm; Leica DM5500 B microscope). Stomata were automatically identified using StomataCounter (www.stomata.science; Fetter, Eberhardt, Barclay, Wing, & Keller, 2019), and identifications were manually corrected to get the final count (stomata mm^−2^). For vein density, a leaf punch of 1.58 cm was taken from the same leaf and stored in a paraformaldehyde–glutaraldehyde fixative solution until analysis. Leaf punches were then prepared following Pérez‐Harguindeguy et al. (2013). Samples were first soaked in 5% NaOH for 4 to 7 days at 60°C until transparent and thereafter stored in 50% ethanol. Samples were then rinsed in water, bleached in 14% NaClO, rinsed in water, stained in 5% safranin red, rinsed in water, and destained in 50% ethanol (each step lasted 5 min). Veins were imaged using the same microscope as above (pixel size: 0.582 µm), capturing at least 8 mm^2^ of leaf area through multiple stitched images. Vein density was measured in ImageJ by thresholding the stitched image to create a binary image of the veins, then generating a skeleton of the veins and analyzing the skeleton using BoneJ's Analyse Skeleton function (Doube et al., 2010). This semi‐automated method generated comparable results compared to hand measurement (data not shown).

### 2.4 Statistical analysis

#### 2.4.1 Testing for general patterns of local adaptation

First, we fitted a general linear model between growth traits (dbh and height) and distance between trial site climate and provenance climate following the quadratic regression model equation

(1) $$Y_{i}=\beta_{0}+\beta_{1}D+\beta_{2}D^{2}+e_{i}$$

where *Y* is dbh or height of the i*th* provenance in a common garden, β_0_ is the intercept, β_1_ and β_2_ are regression coefficients, *D* is the transfer distance between site climate and seed source climate, and *e* is the residual variance. For calculating *D*, we used the differences between provenance origin and trial site location for mean annual temperature (MAT) and mean annual precipitation (MAP), respectively (i.e., positive *D* values indicate that provenances were transferred into colder/drier environments, negative values indicate that provenances were transferred to warmer/wetter conditions). Both climatic variables (MAT and MAP) had been shown to explain a considerable amount of variation in tree growth in earlier studies (e.g., Chakraborty et al., 2016; Wang, Hamann, Yanchuk, O'neill, & Aitken, 2006). This was done separately for each of the three test sites.

#### 2.4.2 Trait divergence among provenances and Q_ST_

We estimated whether provenances or climatic clusters are genetically differentiated in their growth (height and dbh) by applying Q_ST_, a measure of quantitative genetic differentiation that estimates the proportion of genetic variation in a trait among populations relative to the total amount of variation (Leinonen, McCairns, O'Hara, & Merilä, 2013). Q_ST_ is similar to the widely used F_ST_ (Wright, 1949) but takes into account only quantitative trait information without allelic variation at specific loci as in the case of F_ST_. Q_ST_ was calculated as

(2) $$Q_{\text{ST}}=\sigma_{\text{Pop}}^{2}/\left(\sigma_{\text{Pop}}^{2}+2\sigma_{a}^{2}\right)$$

where $\sigma_{\text{Pop}}^{2}$ is the variance among provenances or clusters, respectively, and $\sigma_{a}^{2}$ is the additive genetic variance of a trait obtained from the relatedness among half‐siblings of the same mother tree. We used the method developed by Gilbert and Whitlock (2015), which is implemented in the Q_ST_F_ST_Comp package in R (github.com/kjgilbert/QstFstComp), but calculated only Q_ST_ and its 95% confidence intervals without comparing Q_ST_ to F_ST_, since no neutral diploid markers were available in this study. Q_ST_ was determined across all sites as well as separately for each site.

#### 2.4.3 Additive genetic component and heritability

We estimated additive genetic effects in growth traits by calculating the narrow‐sense heritability (h^2^) across and within provenances from a mixed model of the form:

(3) Y=Xβ+Zp+Zb+Za+e

for across‐provenance heritability within sites (h^2^_site_, n=3330) and

(4) Y=Xβ+Zb+Za+e

for within‐provenance heritability within sites (h^2^_prov_, *n* = 330) with β being a vector of fixed effects (intercept), and *p*, *b*, and *a* random vectors of provenance (climatic cluster), block, and additive genetic effects, respectively. X and Z are incidence matrices assigning fixed and random effects to phenotypic observations in vector Y. Provenance (or climatic cluster) and block effects follow x ~ *N*(0,σ_p,b_^2^) with σ_p_,_b_^2^ being the Provenance (Cluster), or block variance, respectively. Individual‐tree additive genetic effects follow a ~ *N*(0, σ_a_^2^A) where σ_a_^2^ is the additive variance and A the relationship matrix derived from a half‐sib family structure of open‐pollinated mother trees. In this model, we assumed that none of the progenies were full‐siblings, since previous studies have shown that the proportion of full‐siblings in wind‐pollinated trees sampled in forest stands is usually very small and has only little influence on the estimated additive genetic variance (e.g., Bacilieri, Ducousso, Petit, & Kremer, 1996; Kjær, McKinney, Nielsen, Hansen, & Hansen, 2012). Variance components were calculated using an animal model approach (Henderson, 1984; Wilson et al., 2010). The narrow‐sense heritability was calculated as

(5) $$h2=\sigma_{a}^{2}/\left(\sigma_{a}^{2}+\sigma_{e}^{2}\right)$$

where $\sigma_{a}^{2}$ and $\sigma_{e}^{2}$ are the additive and residual variances, respectively. We employed the R package *BreedR* (version 0.12‐4; github.com/famuvie/breedR) which uses a restricted maximum likelihood (REML)‐based variance estimator procedure allowing to infer random genetic effects at individual level. We used the average information matrix (function *ai*), which simulates standard errors from the asymptotic Gaussian joint sampling distribution, to estimate mean and standard errors of variance components. We considered the heritability estimate to be significant, when the lower bound of the 95% confidence interval for heritability was greater than 0.

#### 2.4.4 Variation in phenotypic plasticity of growth traits (GxE)

To test for variation in phenotypic plasticity (i.e., genotype‐by‐environment interactions) and to estimate its contribution to overall phenotypic variation, we formulated a mixed model as follows for height and dbh:

(6) $$Y_{\mathrm{ijklm}}=\beta_{0}+\beta_{1}S_{i}+\beta_{2}P_{j}+\beta_{3}M_{k}\left(P_{j}\right)+\beta_{4}B_{1}\left(S_{i}\right)+\beta_{5}\left(S_{i}P_{j}\right)+\beta_{6}\left(S_{i}M_{k}\left(P_{j}\right)\right)+e_{\text{ijklm}}$$

With Y_ijklm_ being the phenotype of the m*th* tree, belonging to the l*th* block nested within the i*th* site (B_l_(S_i_)), belonging to the k*th* mother tree nested within provenance *j* (M_k_(P_j_), originating from provenance *j* (P_j_) and growing in the i*th* trial site (S_i_). e*_ijklm_* is a random error term, and S_i_P_j_ and S_i_M_k_(P_j_) are the crossed genotype × environment interaction terms separated for provenance‐by‐site and family‐by‐site interactions, respectively. Variance components were expressed as ratios relative to the total phenotypic variation as a percentage of variance explained by the single equation terms, and for this purpose only, all terms were treated as random effects in the model following x ~ *N*(0,σ_x_^2^) with × being the single predictors, respectively. In order to test whether variation in plasticity was uniform among families, we used BLUPs (best linear unbiased predictions) for the GxE family‐interaction term as predicted by the model in equation (6) and calculated the ecovalence (i.e., stability of families across environments) according to Wricke (1962) as follows:

(7) $$W_{i}^{2}=\sum {\left(X_{\text{ij}}-{\bar{X}}_{i.}-{\bar{X}}_{\text{.j}}+{\bar{X}}_{..}\right)}^{2}$$

where *X*_ij_ is the observed trait of family *i* in environment *j*, *X̅*_i._ is the mean trait across families, *X̅*_.j_ is the mean trait across environments, and *X̅*_.._ is the grand mean. Ecovalence was expressed as ratio between family sum of squares (ss_fam_) divided by the total sum of squares across all families, where higher values indicate more plastic genotypes. We used an arbitrary threshold of 0.05 to define extraordinarily plastic genotypes and assigned extraordinary families to provenances in order to see whether they occur more frequently in some environments compared to others. Since our design does not explicitly allow for testing whether plasticity is heritable and therefore adaptive, we used this information just as a broad surrogate.

#### 2.4.5 Intra‐specific variation in functional traits

Since our dataset for functional traits was much smaller compared to growth traits (270 versus 9,990), we used an ANOVA (analysis of variance) approach and treated site, provenance, and provenance‐by‐site as main effects and family as random effect in a linear mixed‐effect model using the *lme4* package in R. Given the limited number of trees that could be measured for functional traits, we decided to capture trait variation at the provenance level rather than at the family level by sampling a larger number of mother trees within provenances and test sites but not to replicate mother trees within site. We used Pearson‐moment correlation and correlated functional and growth traits at the individual‐tree level (tree‐wise functional trait versus growth) as well as at the provenance level (provenance mean functional trait versus single‐tree growth) to test whether functional traits can be used to select more vigorous or resilient trees. Finally, we calculated the summer heat:moisture index (SHM), an index used to describe the long‐term drought regime in seed zones (Wang, Hamann, Spittlehouse, & Murdock, 2012), to compare mean functional trait values to climate at seed origin of provenances to test for adaptive patterns in functional trait variation

(8) SHM=MWMT/(MSP/1,000)

where MWMT is the mean temprature of the warmest month in °C, and MSP the mean summer precipitation (May to September) in mm. Higher SHM values indicate drier climatic conditions. We used different climatic variables for assessing intra‐specific differences in growth (MAT) and in functional traits (SHM) in order to account for the fact that genecological differences in tree growth in many earlier studies were best explained by the average temperature regime (e.g., Wang, O'Neill, & Aitken, 2010; Jobbágy & Jackson 2000; Loehle, 1998), whereas adaptive differences in functional traits with importance for drought adaptation were best explained by climate variables indicating probability of drought occurrence (e.g., Lamy et al., 2014; Rungwattana et al., 2018). We calculated a linear model between the functional trait value at provenance level and the climatic variables at seed origin and reported slopes and p‐values separately for the three test sites. *p*‐Values were corrected for multiple comparisons by applying a Benjamini–Hochberg adjustment procedure (Benjamini & Hochberg 1995).

## 3 RESULTS

### 3.1 Site and provenance characteristics

**FIGURE 1:**
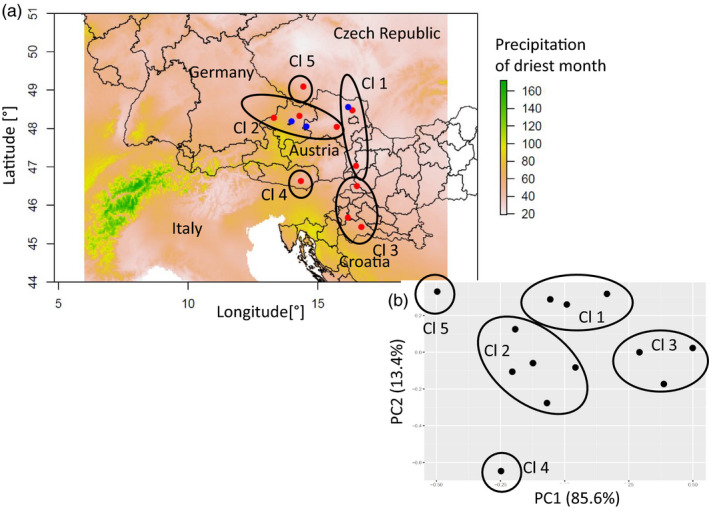
Geographic origin and climate of provenances and location of trial sites. (a) Red dots show provenances, and blue dots show trial sites. Color displays precipitation of driest month in mm. (b) First two principal components of climatic variables that were used for assigning provenances to climatic clusters. Higher PC1 values indicate higher temperatures, whereas higher PC2 values indicate lower precipitation

Using principal component analysis, provenances were assigned to five climatic clusters (Figure 1b), with the first principal component axis being clearly related to temperature variables (85.6% of explained variation), whereas the second principal component axis corresponded to precipitation regime (mean annual precipitation and mean summer precipitation; 13.4% explained variation). Cluster 1 consists of the two provenances from the northeast and southeast of Austria with stronger continentality and more frequent summer drought compared to the rest. Cluster 2 contains provenances from northern Austria (1, 2, and 8) characterized by a stronger Atlantic influence with lower mean annual temperature and a lower probability of summer drought occurrence. The three provenances from Slovenia and Croatia together were assigned to Cluster 3 with warmer mean annual temperature (0.6°C–1.6°C above average). Finally, provenances 14 (southern Austria) and 17 (Czech Republic) were assigned to single‐provenance clusters 4 and 5, and the latter was characterized by colder mean growing conditions of about −1.8°C compared to the overall mean.

Survival rate after 10 years was 94% (9,306 living trees), and mortality did not significantly differ between sites nor between provenances. As expected, mean height and dbh after 10 years were higher at the moist site (height: 5.6 m; dbh: 5.4 cm) and lower at the intermediate (height: 4.5 m; dbh: 4.1 cm) and dry sites (height: 4.3 m; dbh: 3.8 cm).

### 3.2 Local adaptation of provenances and Q_ST_

**FIGURE 2:**
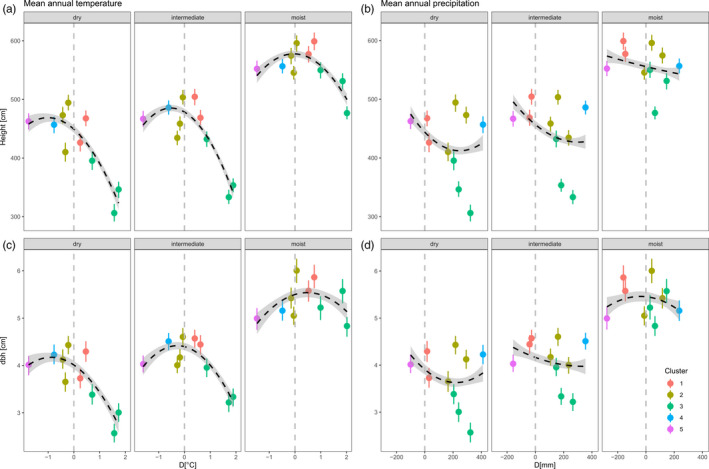
Provenance and cluster differentiation for height (a) and dbh (b) as a function of temperature (a,b) and precipitation (c,d) transfer distance, which is the difference between mean annual temperature/precipitation at test site and provenance origin. Positive values indicate Tprov > Tsite, and negative values indicate Tprov < Tsite. Vertical lines show standard errors. Dashed line shows where the transfer distance is 0 (i.e., provenance climate = site climate)

**FIGURE 3:**
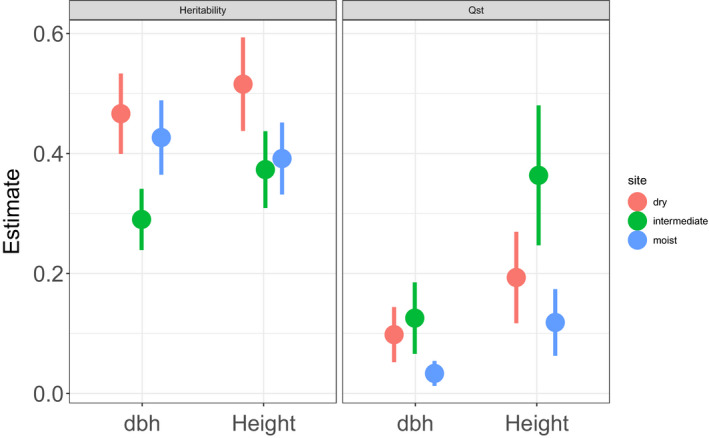
Heritability and QST for height and dbh calculated for each site separately (red, green, blue). Error bars display standard errors

Nonlinear models with the temperature transfer distance as quadratic term were highly significant in all three test sites (Table 2) and explained between 5% (moist site) and 14% (intermediate site) of the overall variation. Clusters 1, 2, and 4 performed best with growth decreasing toward both colder and warmer provenance climates (Figure 2). The local maximum for height and dbh coincided with a temperature distance of approximately 0°C at the moist site, but shifted toward colder provenance climates in the intermediate and dry sites (i.e., cold cluster 2 increased growth toward drier conditions compared to the warm cluster 3, Figure 2a,b). Differences in mean annual precipitation between provenance origin and trial site explained less variation compared to MAT, and a classical bell‐shaped response curve could not be revealed in most cases (Figure 2c,d). Overall, Q_ST_ was significantly different from zero for growth and higher for height growth (mean: 0.235, 95% CI: 0.074–0.412) than for dbh (mean: 0.07, 95% CI: 0.017–0.145). Congruently with the results from the temperature distance model above, mean Q_ST_ was higher in the intermediate and dry sites (height‐Q_ST_: 0.365 and 0.193; dbh‐Q_ST_: 0.126 and 0.098, respectively) and significantly lower at the moist site (height‐Q_ST_: 0.119, dbh‐Q_ST_: 0.033, the 95% CIs did not overlap between dry and intermediate site; Figure 3).

**TABLE 2:** Summary statistics of the climatic transfer model (equation 1)

Trait	Site	Mean annual temperature					Mean annual precipitation				
		Coefficient	Estimate	t‐value	*p*‐value	R^2^	Coefficient	Estimate	t‐value	*p*‐value	R^2^
Height	WB (dry)	Intercept	449.19	143.39	<.001	0.12	Intercept	444.00	111.779	<.001	0.02
		D[°C]	−39.03	−15.96	<.001		D [mm]	−0.26	−6.863	<.001	
		D^2^ [°C]	−19.35	−9.59	<.001		D^2^ [mm]	0.00	4.726	<.001	
	WS (intermediate)	Intercept	478.47	173.78	<.001	0.14	Intercept	456.80	141.221	<.001	0.03
		D[°C]	−25.92	−11.16	<.001		D [mm]	−0.20	−8.177	<.001	
		D^2^ [°C]	−24.79	−13.93	<.001		D^2^ [mm]	0.00	3.285	<.01	
	WL (moist)	Intercept	577.68	207.05	<.001	0.05	Intercept	555.70	181.059	<.001	0.01
		D[°C]	−1.86	−0.72	n.s.		D [mm]	−0.06	−3.745	<.001	
		D^2^ [°C]	−18.26	−10.05	<.001		D^2^ [mm]	0.00	0.24	n.s.	
Diameter at breast height (dbh)	WB (dry)	Intercept	4.01	90.73	<.001	0.06	Intercept	3.91	71.585	<.001	0.01
		D[°C]	−0.369	−10.69	<.001		D [mm]	0.00	−4.912	<.001	
		D^2^ [°C]	−0.209	−7.35	<.001		D^2^ [mm]	0.00	3.783	<.001	
	WS (intermediate)	Intercept	4.398	110.23	<.001	0.05	Intercept	4.17	92.384	<.001	0.00
		D[°C]	−0.14	−4.16	<.001		D [mm]	0.00	−3.299	<.001	
		D^2^ [°C]	−0.252	−9.77	<.001		D^2^ [mm]	0.00	1.171	n.s.	
	WL (moist)	Intercept	5.50	0.049	<.001	0.009	Intercept	5.46	102.679	<.001	0.00
		D[°C]	0.16	0.05	<.001		D [mm]	0.00	−1.15	n.s.	
		D^2^ [°C]	−0.171	0.03	<.001		D^2^ [mm]	0.00	−2.396	<.05	

Note

Significance levels are as follows: *:*p* < .05; ***:*p* < .01; ***:*p* < .001.

Abbreviation: n.s., not significant.

### 3.3 Additive variance and narrow‐sense heritability (h^2^)

Additive genetic variance was highly significant for height and dbh when calculated across provenances within sites (h^2^_site_) and remained also significant within most provenances within sites (h^2^_prov_) despite the much lower sample size (3,330 versus 330; Table 3). Narrow‐sense heritability reached values up to 0.64 (provenance 2, dry site) for height and 0.68 for dbh (provenance 12, dry site). Narrow‐sense heritability was significantly higher for both height and dbh at the dry test site (95% CI_Height_ = 0.438–0.594; 95% CI_dbh_ = 0.399–0.534) compared to the intermediate site (95% CI_Height_ = 0.309–0.437; 95% CI_dbh_ = 0.239–0.341), but did not significantly differ between the dry and moist sites nor between the intermediate and moist sites (Figure 3).

**TABLE 3:** Narrow‐sense heritability estimates (h^2^) for height and dbh within sites (h^2^_site_) and within provenances within sites (h^2^_prov_)

	Site	WB = dry		WS = intermediate		WE = moist	
	Trait	Height	dbh	Height	dbh	Height	dbh
	**Overall h^2^_site_; *n* = 3,330**	**0.516 (0.438–0.594)**	**0.467 (0.399–0.534)**	**0.373 (0.309–0.437)**	**0.29 (0.239–0.341)**	**0.392 (0.332–0.452)**	**0.427 (0.365–0.489)**
h^2^_prov_; *n* = 330	Geinberg	**0.519 (0.09–0.948)**	**0.617 (0.147–1.00)**	0.144 (0.0–0.38)	0.251 (0.00–0.544)	**0.448 (0.05–0.847)**	**0.436 (0.04–0.832)**
	Linz	**0.638 (0.162–1.00)**	**0.556 (0.113–0.998)**	0.629 (0.0–1.00)	**0.662 (0.161–1.00)**	0.351 (0.00–6.97E−01)	**0.542 (0.316–0.768)**
	Rainfeld	**0.451 (0.04–0.863)**	**0.486 (0.06–0.911)**	**0.415 (0.03–0.793)**	**0.399 (0.03–0.767)**	0.262 (0.0–0.57)	**0.555 (0.112–0.997)**
	Braunsberger Wald	0.32 (0.0–0.671)	0.289 (0.0–0.626)	0.18 (0.0–0.439)	0.035 (0.00–0.216)	**0.534 (0.08–0.99)**	**0.593 (0.112–1.00)**
	Luising	**0.588 (0.128–1.00)**	**0.676 (0.182–1.00)**	0.298 (0.0–1.0)	0.262 (0.00–0.564)	0.141 (0.0–0.421)	0.262 (0.00–0.582)
	Klagenfurt	**0.574 (0.128–1.00)**	**0.637 (0.164–1.00)**	0.143 (0.0–1.00)	0.217 (0.00–0.492)	**0.496 (0.08–0.912)**	**0.514 (0.09–0.939)**
	Hluboka (CZ)	0.067 (0.0–0.277)	0.097 (0.00–0.329)	0.115 (0.0–0.34)	0.164 (0.00–0.394)	0 (0.0–1.0)	0.082 (0.00–0.301)
	Kutina (HR)	0.341 (0.0–0.7)	0.36 (0.00–0.728)	0.195 (0.0–0.47)	0.23 (0.00–0.442)	0.167 (0.0–0.433)	0.155 (0.00–0.416)
	Velika Gorica (HR)	0.046 (0.0–0.26)	0 (0.00–6.38E−06)	0.04 (0.0–0.23)	0 (0.00–1.323E−06)	0 (0.0–1.00)	0 (0.00–4.16E−06)
	Murska suma (SLO)	**0.425 (0.03–0.82)**	**0.377 (0.01–0.748)**	0.184 (0.0–0.44)	**0.4 (0.03–0.555)**	**0.586 (0.132–1.00)**	**0.562 (0.117–1.00)**

Note

Bold values show significant h2 estimates; 95% CI given in parentheses.

### 3.4 Variation in plasticity of growth (GxE)

Both GxE terms (provenance‐by‐site and family‐by‐site) were significant for height and dbh, but explained only a minor proportion of the overall variance when compared to the remaining terms (Table 4). As such, GxE terms explained 4% of total height variation (2.75% attributable to provenance × site, 1.25% attributable to family × site) and 3.8% for dbh variation (2% attributable to provenance × site, 1.75% explained by family × site).

**TABLE 4:** Summary statistics from the mixed linear model (equation 5) and relative variance explained by each predictor for height and dbh

Trait	Predictor	Variance	se	% of variance explained
Height	site	4,999.1	5,067.3	22.05
	provenance	2067.1	1,103.9	**9.12**
	family	896.73	132.29	**3.96**
	block	58.52	62.95	0.26
	prov × site	623.45	227.3	**2.75**
	fam × site	283.55	87.97	**1.25**
	residual	13,743	208.78	**60.62**
dbh	site	0.7257	0.73647	15.91
	provenance	0.13028	0.08454	**2.86**
	family	0.24505	0.035	**5.37**
	block	0.009874	0.01093	0.22
	prov × site	0.093256	0.03596	**2.04**
	fam × site	0.079982	0.02186	**1.75**
	residual	3.2761	0.04977	**71.84**

Note

Values in bold show significant components.

Abbreviation: se, standard error.

**FIGURE 4:**
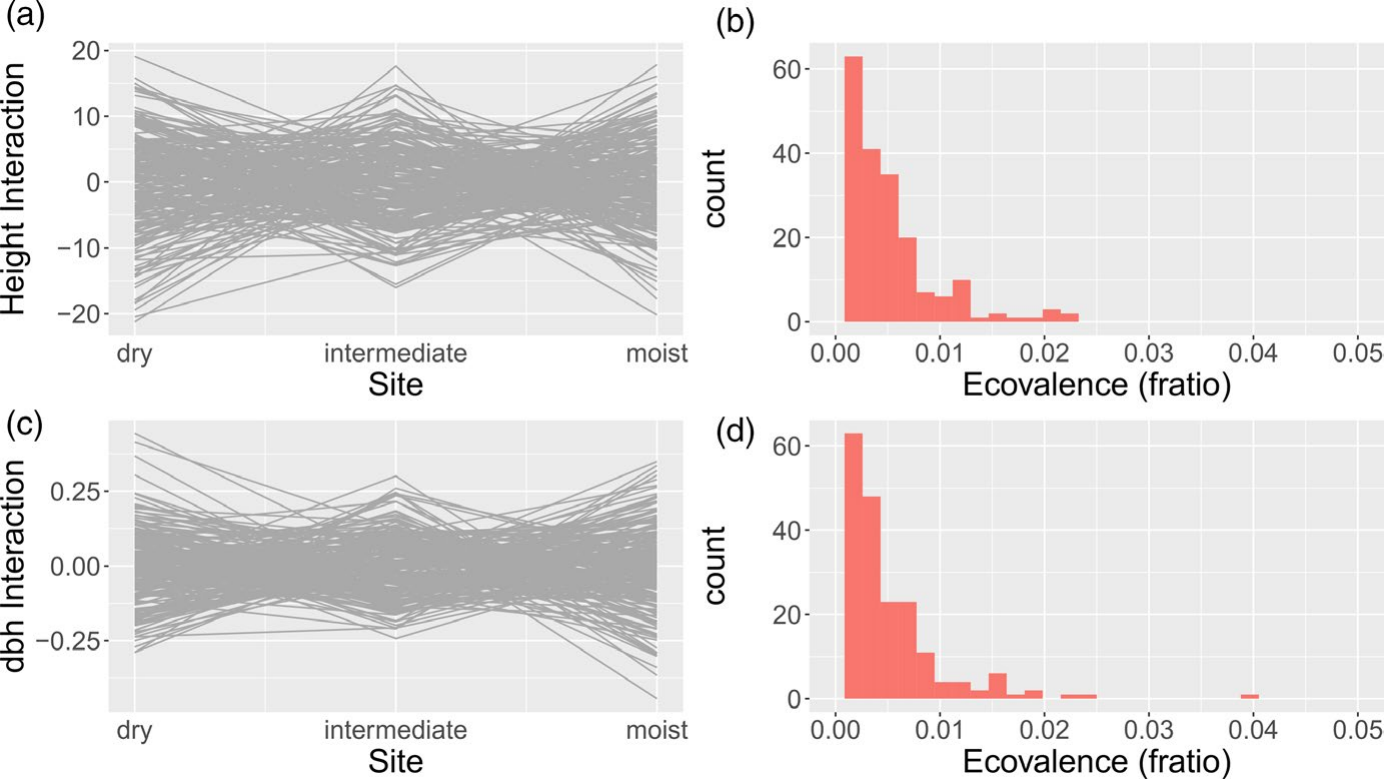
Genotype‐by‐environment interactions for height growth (a,b) and dbh (c,d). Interactions on the Y‐axis are given as BLUPs of interactions. Histograms in c) and d) show uniformity in plasticity among families expressed as ecovalence (fratio) counts

There were no differences depending on whether provenance or climatic cluster was used as covariate. In comparison, site alone explained approximately 22% of the phenotypic variation for height and 16% for dbh, while provenance explained 9% of the variation for height and 3% for dbh (Table 4). Ecovalence of families was in general low with values fluctuating between 2.8E‐05–0.028 for height and 2.6E‐05–0.041 for dbh, and no family was characterized as extraordinarily plastic (Figure 4).

### 3.5 Variation in functional traits, correlation with seed source climate, and relation to growth traits

**FIGURE 5:**
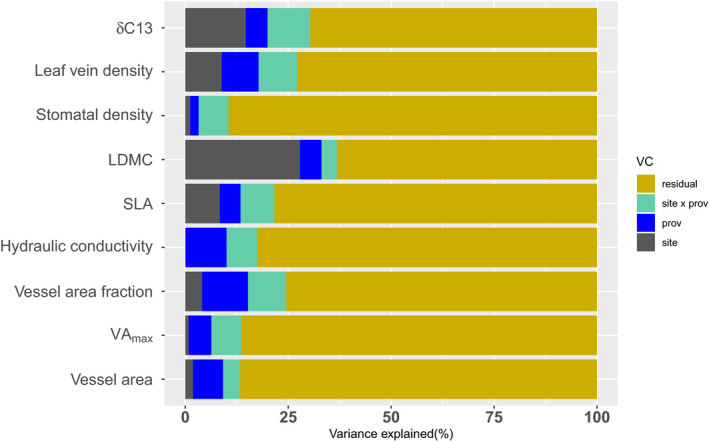
Fraction of variance explained by site, provenance, provenance‐by‐site, and residuals for leaf and wood functional traits

Functional traits varied significantly among sites (*p* < .001 for SLA, LDMC, Leaf vein density, and δC^13^; *p* < .01 for vessel area fraction), but also significantly among provenances (*p* < .001 for vessel area fraction; *p* < .01 for leaf vein density and hydraulic conductivity; *p* < .05 for vessel area). Significant provenance‐by‐site interactions appeared only in δC^13^ (*p* < .05). Relative variance proportions explained by the three predictors are presented in Figure 5.

**FIGURE 6:**
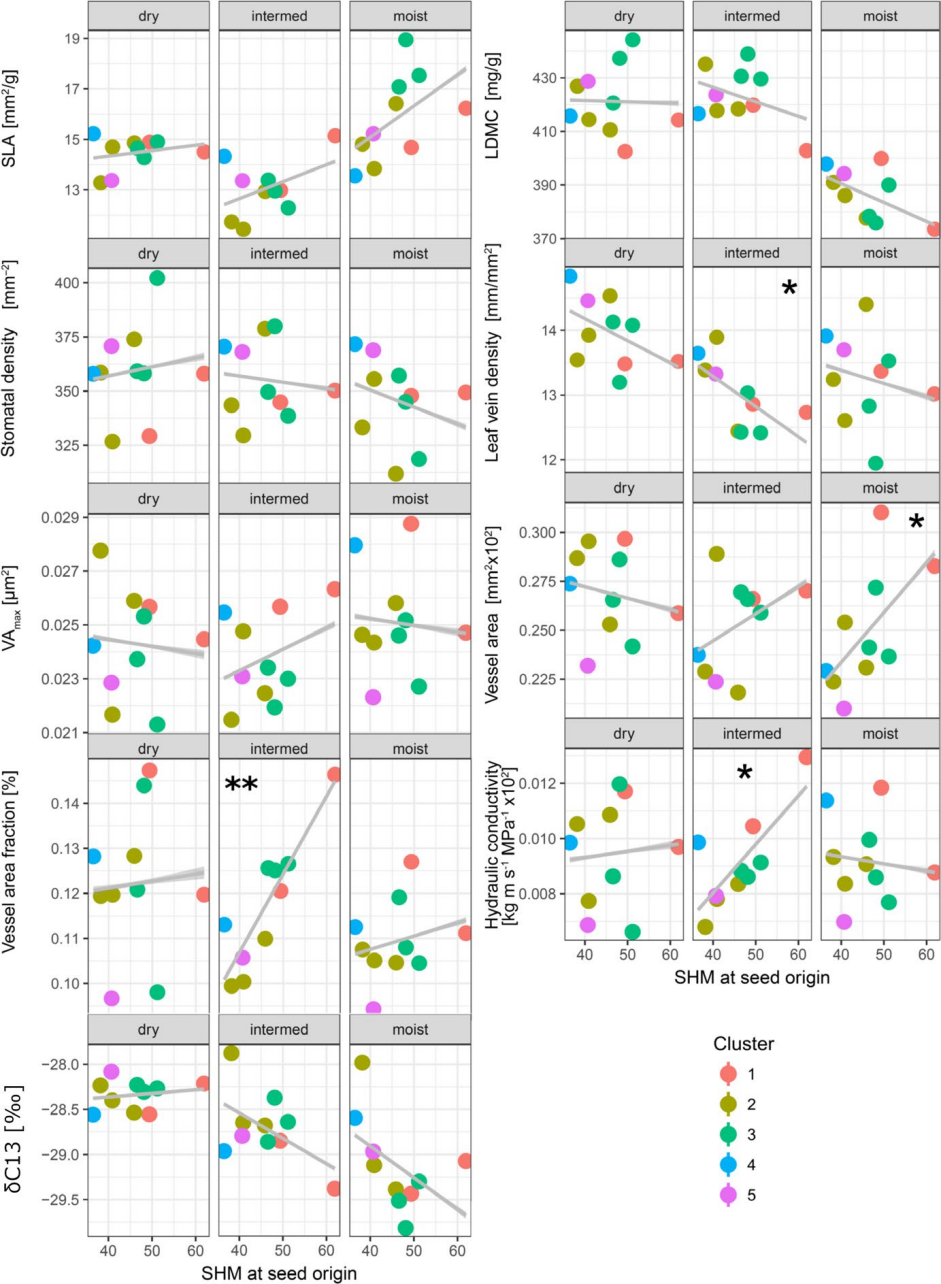
Relationship between functional traits at provenance level and source climate expressed as summer heat:moisture index (SHM). Asterisks show significant associations after adjustment for multiple testing

Congruently with the results above, correlations between dryness at seed origin (SHM) and functional traits at provenance level varied strongly among sites: Provenances from drier regions had significantly lower leaf vein density (at the intermediate site), a higher mean vessel area (moist site), higher hydraulic conductivity (intermediate site), and a higher vessel area fraction (intermediate site). A trend in more negative δC^13^ values was observed for provenances from the dry and warm clusters (intermediate and moist site). At the dry site, however, no significant relationship between seed source climate and functional trait variation was observed (Figure 6).

**FIGURE 7:**
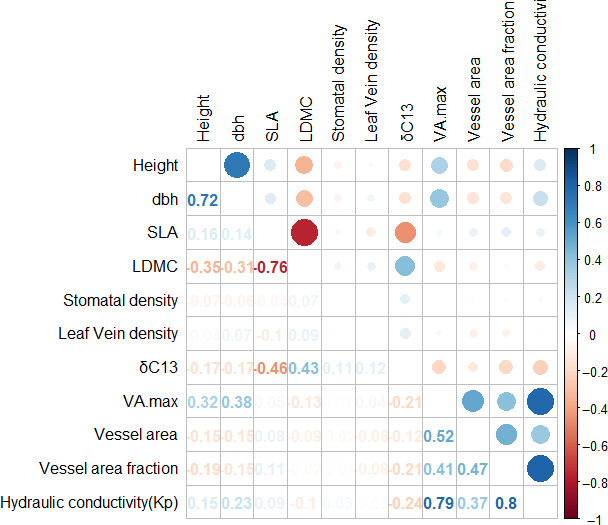
Correlations between growth traits and functional traits expressed as Pearson‐moment correlations

We found strong and significant phenotypic correlations among traits that belong to the same trait category (leaf or wood), but only moderate evidence for relations between functional traits and growth traits. Among those, tree height was strongest negatively related to leaf dry matter content with r = −0.35. Dbh was strongly correlated to VA_max_ (r = 0.358). For both trait combinations, correlation coefficients were highest at the intermediate site and lower at dry and moist sites when calculated site‐wise (Figure 7 and Figures S2–S9).

## 4 DISCUSSION

Trees can potentially respond to environmental selection pressure in three different ways: by migrating to more suitable growing sites, by directional selection within populations with the preferential survival of outlier phenotypes, or by adjusting their phenotypes under novel environmental conditions through phenotypic plasticity. In this study, we disentangled these three potential pathways in order to evaluate which of the scenarios will be most likely for pedunculate oak, an important temperate tree species in Europe which was shown to be vulnerable under increasing drought in the near future (Levanič, Čater, & McDowell, 2011). All three outcomes (migration, selection, and plasticity) have statistical counterparts that were employed in our study: Differentiation among populations along an ecological transfer distance, that is, climate, as well as Q_ST_, can be seen as indicators of local adaptation to home temperature regimes (Kawecki & Ebert, 2004; Sáenz‐Romero et al., 2017). Second, additive genetic variation and significant narrow‐sense heritability imply that directional selection has the capacity to drive adaptation to novel climate conditions and may be utilized in breeding for more resilient genotypes (Harfouche et al., 2012). Finally, when some genotypes are more plastic than others, GxE can become an important evolutionary feature and potentially drive adaptation to novel environments assuming that the phenotypic change is not maladaptive and that plasticity itself has a heritable basis (Pigliucci, 2005).

### 4.1 Local adaptation of provenances and quantitative trait differentiation

We used growth expressed as height and diameter at breast height after 10 years as a strong surrogate for fitness, which is a reasonable assumption given that larger trees compete more effectively for light and will more likely survive density‐dependent competition during the adolescent growing stage (Aitken & Bemmels, 2016; Alberto et al., 2013). We observed a clear pattern of local adaptation of provenances for mean annual temperature at all three sites resulting in decreasing dbh and height growth with increasing temperature transfer distance. Consequently, local seed stands (clusters 1 and 2) are still better adapted under current climatic conditions compared to “warmer” or “colder” provenances. In contrast, local adaptation of provenances was less significant for distance between moisture regimes expressed as mean annual precipitation. Replacing mean annual precipitation by mean summer precipitation or summer heat:moisture index resulted in the same pattern (data not shown). A reasonable explanation for this finding is that some provenances obviously can benefit from warmer growing conditions at intermediate and dry sites (relative to the local provenance) without facing restrictions of less available moisture. This is particularly true for provenances from clusters 2 and 4 which originated from colder and wetter growing regimes (Austria‐north and Austria‐south). Unlike these clusters, provenances from southern regions (cluster 3: Croatia, Slovenia) experienced a significant reduction in both temperature and moisture when transferred to intermediate and dry trial sites. Based on these findings, it seems likely that, at regional scale, temperature is a more important evolutionary driver of adaptation in pedunculate oak than moisture. This makes sense, given that late frost events in spring are likely to occur at all the three trial sites and that provenances from the southern cluster originate from regions with comparably mild winters (see mean coldest month temperatures in Table 1). This could explain the strong observed signal of local adaptation for mean annual temperature, since both climatic variables are highly correlated in our dataset (r = 0.91). Hence, actual seed transfer guidelines for forest reproductive material in Europe seem to be appropriate when recommending the use of local against foreign seeds. Ignoring such guidelines could lead to a loss in mean height after 10 years of approximately 2 meters at dry sites when the worst and best provenances in Figure 2 are compared. Our results corroborate a study on closely related sessile oak (*Quercus petraea*), which found analog patterns of maladaptation with increasing climatic distance from the provenances' source climate (Sáenz‐Romero et al., 2017). Surprisingly, differentiation among provenances found in our study was similar or even higher compared to Sáenz‐Romero et al. (2017), although provenances in our study originated from across a much smaller geographical range (Saenz‐Romero et al. included populations from southern Norway to northern Turkey). The reasons for this can be that both species have different adaptive capacities or that local adaptation among populations is convergent and reaches a maximum at moderate geographical scales. In any case, our study strongly suggests that a transfer of seed material over a climatic distance of ~2°C from the warmer side of the distribution will potentially result in genetic maladaptation in pedunculate oak under current climate. On the other hand, seed transfer from the colder part of the distribution (e.g., climatic cluster 5) seems to be less problematic under current climate conditions, since the local maximum of the response curve shifted stronger toward negative *D* values from moist to dry sites (Figure 2). While the general and intuitive expectation is that seed material should probably be transferred from warmer to colder regions in order to track the ecological optimum when temperature is expected to rise in the future, our data add an important caveat. One possible reason is that some traits that confer adaptation to colder environments may also be beneficial in dry environments such as a higher resistance against freezing‐induced embolism (Olson et al., 2018).

Generally, Q_ST_ was in accordance with the results above since Q_ST_ was highest at the intermediate site and lower at the moist and dry site (Figure 3 and Table 3; all Q_ST_ results are significantly >0). Our data did not permit to compare Q_ST_ to an estimate of historic gene flow among populations (such as F_ST_) in order to control for effects of neutral genetic drift, as suggested by Gilbert & Whitlock (2015). However, in combination with the response curve in Figure 2, it is very likely that populations of pedunculate oak have experienced spatially divergent selection at a moderate geographical scale and do not show trait divergence because of random genetic drift alone. Moreover, our Q_ST_ estimates for height after 10 years (e.g., 0.36 at the intermediate site) are similar to that for 6‐year height at a regional spatial scale for *Q. petraea* (0.32 in Kremer, Zanetto, & Ducousso, 1997).

### 4.2 Heritability and genotype‐by‐environment interactions in growth traits

Heritability (h^2^) is a measure that quantifies whether or not populations are able to adapt to environmental pressures via directional selection (Falconer & MacKay, 1989). The higher the heritability the higher is the populations' ability to change its mean phenotype toward the new optimum under natural selection (e.g., Kelly, 2011). Additionally, high heritability permits to track the optimum under weaker selection pressure, which reduces the probability of genetic bottlenecks and can therefore avoid loss of genetic diversity due to genetic drift (Lacy, 1987). This is an important aspect given that large‐scale tree mortality after drought events has already become more frequent and will further increase in the near future (Allen et al., 2010). We found high and significant heritability in growth reaching 0.64 for height at the dry trial site, which constitutes a promising basis for breeding programs from large collections of progeny tests. Interestingly, the heritability for height growth was substantially higher at the dry site compared to moist and intermediate sites, suggesting higher prediction accuracy when selecting candidate trees for dry environments under future adaptive forest management. This could in fact be a starting point for future studies aiming at identifying molecular variation at DNA level associated with higher drought tolerance in pedunculate oak.

Genotype‐by‐environment interactions were significant for growth but explained only a relatively small proportion of the phenotypic variation (4%). Plasticity was largely uniform among families and provenances, and ecovalence was generally low (Figure 4b,d). Ahrens et al. (2019) carried out a similar study on *Corymbia calophylla*, which occurs over a steep environmental gradient in Australia, and found no significant genotype‐by‐environment interactions in height growth across a drought gradient in three common gardens. Our results add new evidence that GxE in growth response of trees, regardless whether considered at provenance or family level, is probably less important than was previously hypothesized and will little contribute to climatic adaptation under rapid climate change. However, when discussed in the context of adaptive tree breeding, we nevertheless see the crucial importance of accounting for GxE in the future, since even relatively small GxE effects have the potential to confound heritability estimates and consequently breeding success as was shown in earlier studies (e.g., Chen, Karlsson, & Wu, 2017; Li, Suontama, Burdon, & Dungey, 2017; Raymond, 2011).

### 4.3 Functional trait variation, correlation with seed source climate, and relations between functional traits and growth

Functional traits analyzed in this study are related to drought adaptation of plants, and we therefore tested relationships between functional trait variation, growth, and seed source climate. Unraveling strong adaptive signals of functional trait variation among provenances or strong correlations between functional traits and growth could assist selection of more resilient provenances or genotypes. We found a few strong associations between functional traits and dryness at seed origin, but with varying strength among test sites. Unexpectedly, provenances were poorly differentiated at the dry site for all functional traits, where no significant associations with seed source climate were revealed (Figure 6). This seems to be in broad agreement with findings from Ramírez‐Valiente et al. (2018) who reported a similar inconsistent relationship between functional traits and seed source climate in *Quercus oleoides*. Leaf stable carbon isotope ratio (δ^13^C), as a proxy for water‐use efficiency, was by trend more negative in provenances that originated from drier regions, suggesting that these provenances experienced less drought stress in the intermediate and moist sites. Overall, our results are in concordance with findings of other studies on closely related oak species which found moderate to strong differentiation among provenances for leaf and wood functional traits (Ramírez‐Valiente et al., 2018; Torres‐Ruiz et al., 2019). Consequently, our results are a first hint that *Q. robur* populations have experienced spatially varying selection during postglacial re‐colonization in functional traits at moderate geographical scale.

### 4.4 Assisted migration, breeding, or phenotypic plasticity? How can tree populations adapt to climate change?

Our analysis of intra‐specific variation in this important tree species suggests that adaptive variation (h^2^ and Q_ST_) in growth is stronger than plastic responses (GxE). Although plastic responses have been intensively discussed as a potential evolutionary strategy for trees to avoid mismatches between biological requirements and environmental change (e.g., Corcuera, Cochard, Gil‐Pelegrin, & Notivol, 2011), it is still unclear in the majority of investigated cases whether observed variation in plasticity is adaptive at all or may be simply maladaptive (Matesanz & Valladares, 2014). Moreover, narrow‐sense heritability was significant and high for height and dbh at the dry trial site which may resemble best future climatic conditions in the temperate part of the *Quercus robur* distribution. Consequently, the evidence suggests that directional selection within populations will likely determine the future trajectory of *Quercus robur* under climate change. This has several implications for future adaptive forest management, since the high heritability in growth observed in the dry testing site (see Figure 3) can lead to breeding success and high prediction accuracy when testing candidate trees as potential gene donors capable of tolerating drier conditions. Based on our findings, transfer of genotypes from southern regions such as Croatia to eastern Austria could potentially lead to maladaptation in growth which is most likely caused by lower frost tolerance. Therefore, we believe that the high uncertainty of assisted gene flow combined with the risks associated with these schemes (e.g., Grady, Kolb, Ikeda, & Whitham, 2015) calls for directional selection through tree breeding at moderate geographical scales.

## CONFLICT OF INTEREST

None declared.

## Supporting information

Fig S1

Figs S2–S9

Table S1

## Data Availability

The raw data will be available under the Zenodo.org Digital Repository soon under https://zenodo.org.
