# Supplementary material for: Assessing adaptive and plastic responses in growth and functional traits in a 10‐year‐old common garden experiment with pedunculate oak (Quercus robur L.) suggests that directional selection can drive climatic adaptation
Source: Evol Appl. 2020 Jun 18;13(9):2422–38. doi: 10.1111/eva.13034 (PMC7513705; doi:10.1111/eva.13034)

**Supplementary Fig S1:** Mother tree identifier matrix for provenance #2 (representatively).Numbers in red circles show the same mother tree). Scheme was randomized for each provenance in each cell.


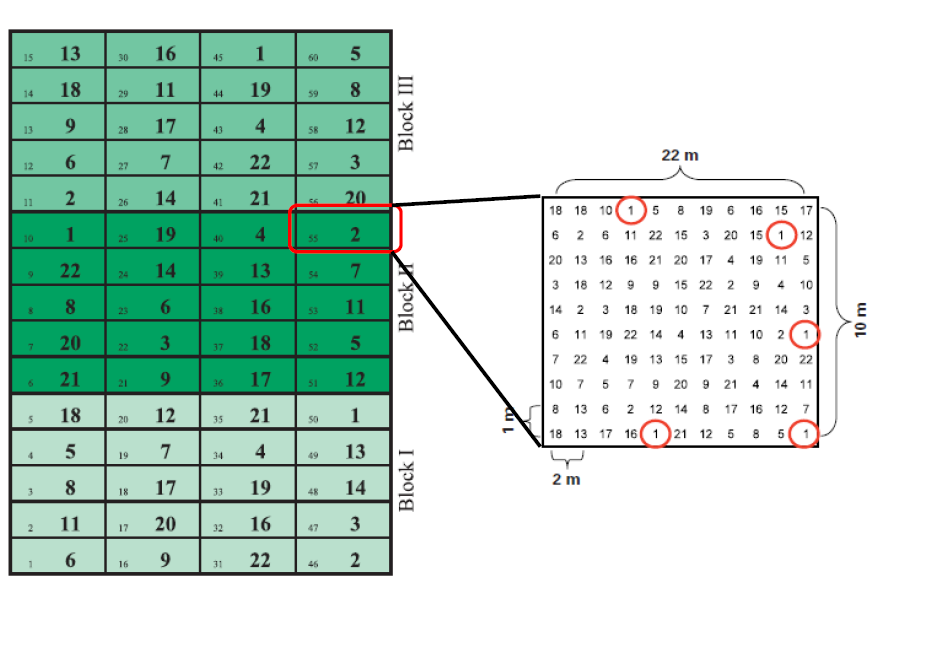

Supplement: Supplementary file 1 — Fig S1 [file EVA-13-2422-s001.doc]
