# Supplementary material for: Assessing adaptive and plastic responses in growth and functional traits in a 10‐year‐old common garden experiment with pedunculate oak (Quercus robur L.) suggests that directional selection can drive climatic adaptation
Source: Evol Appl. 2020 Jun 18;13(9):2422–38. doi: 10.1111/eva.13034 (PMC7513705; doi:10.1111/eva.13034)

**Fig. S2:** Phenotypic correlations between fitness and functional traits (provenance mean (functional)-single tree (fitness) -> **across all sites**

**Fig. S3:** Phenotypic correlations between fitness and functional traits (provenance mean (functional)-single tree (fitness) **-> dry site**


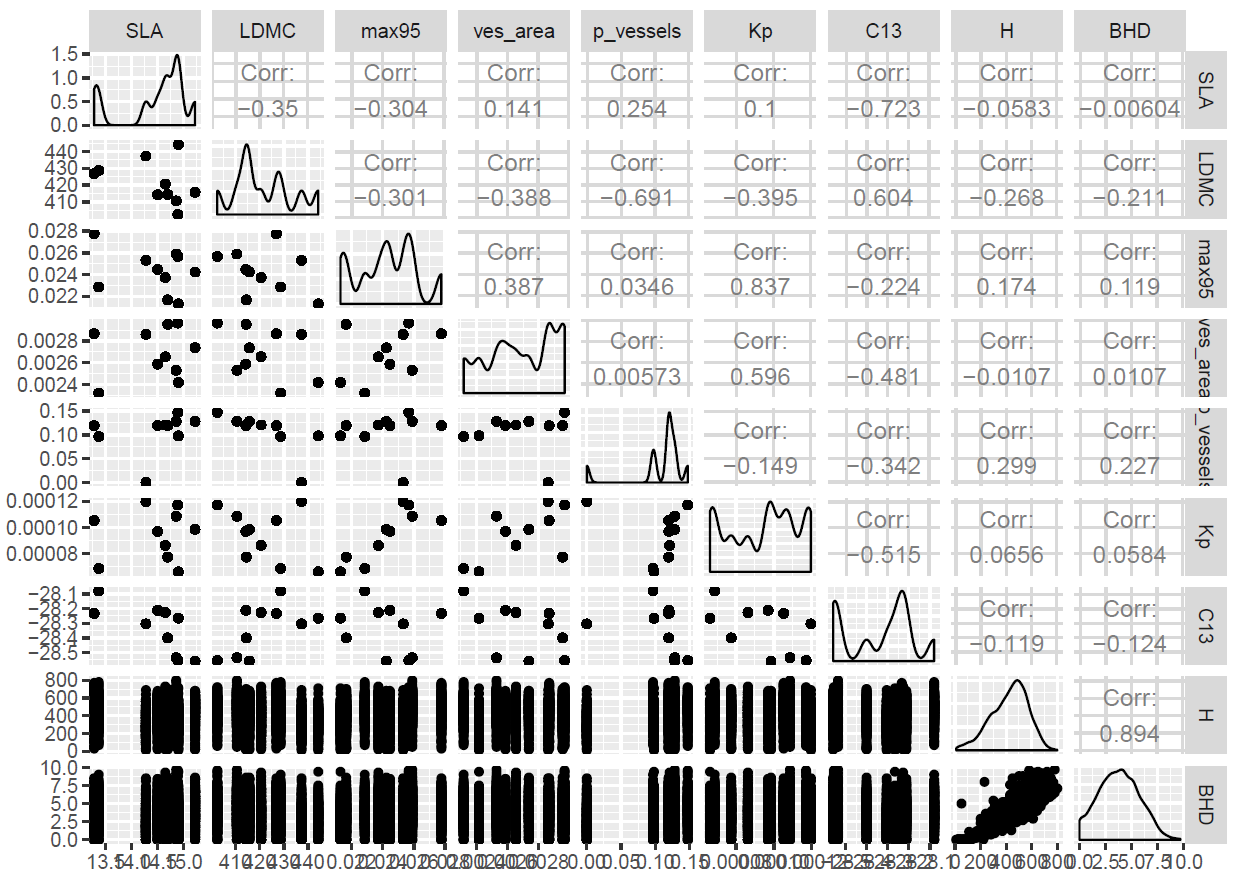


**Fig. S4:** Phenotypic correlations between fitness and functional traits (provenance mean (functional)-single tree (fitness) **-> intermed. site**


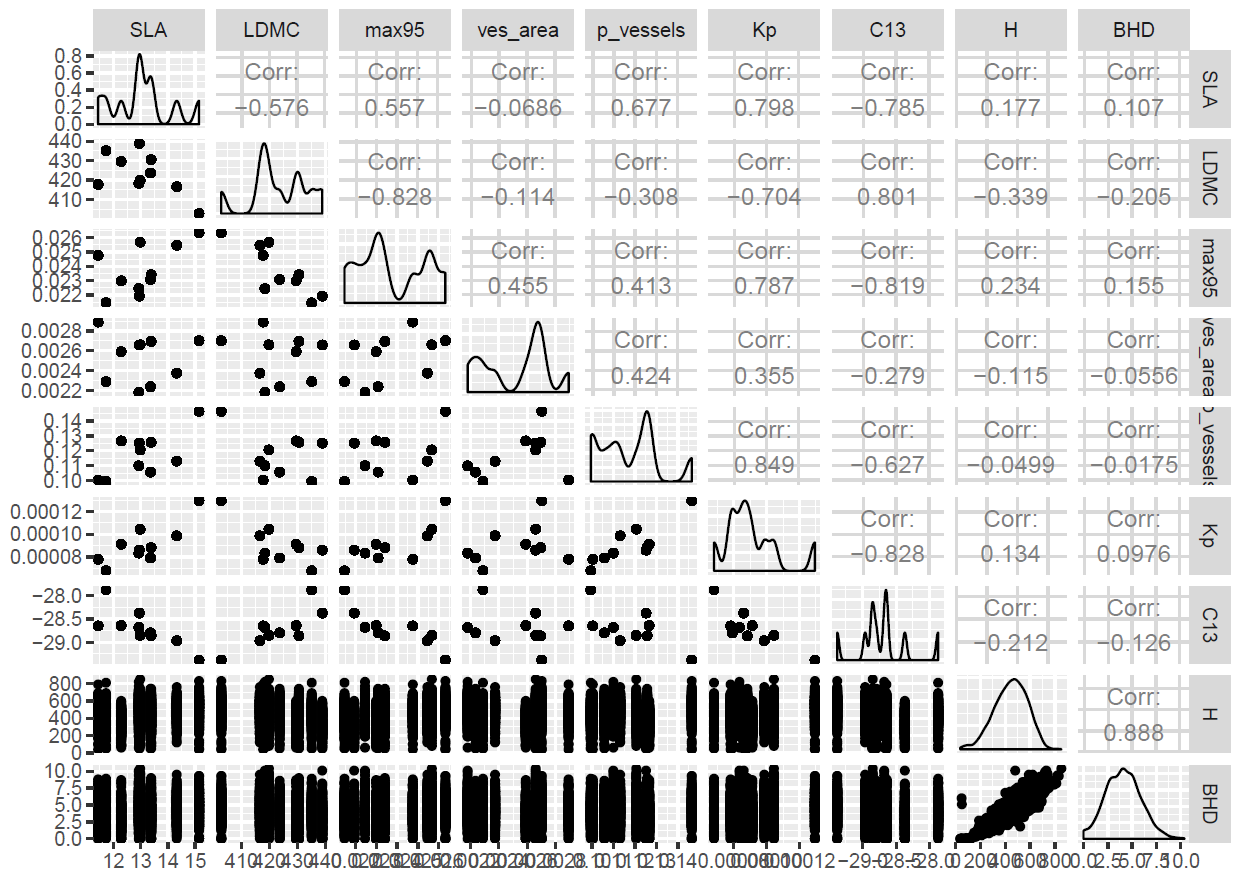


**Fig. S5:** Phenotypic correlations between fitness and functional traits (provenance mean (functional)-single tree (fitness) **-> fresh site**


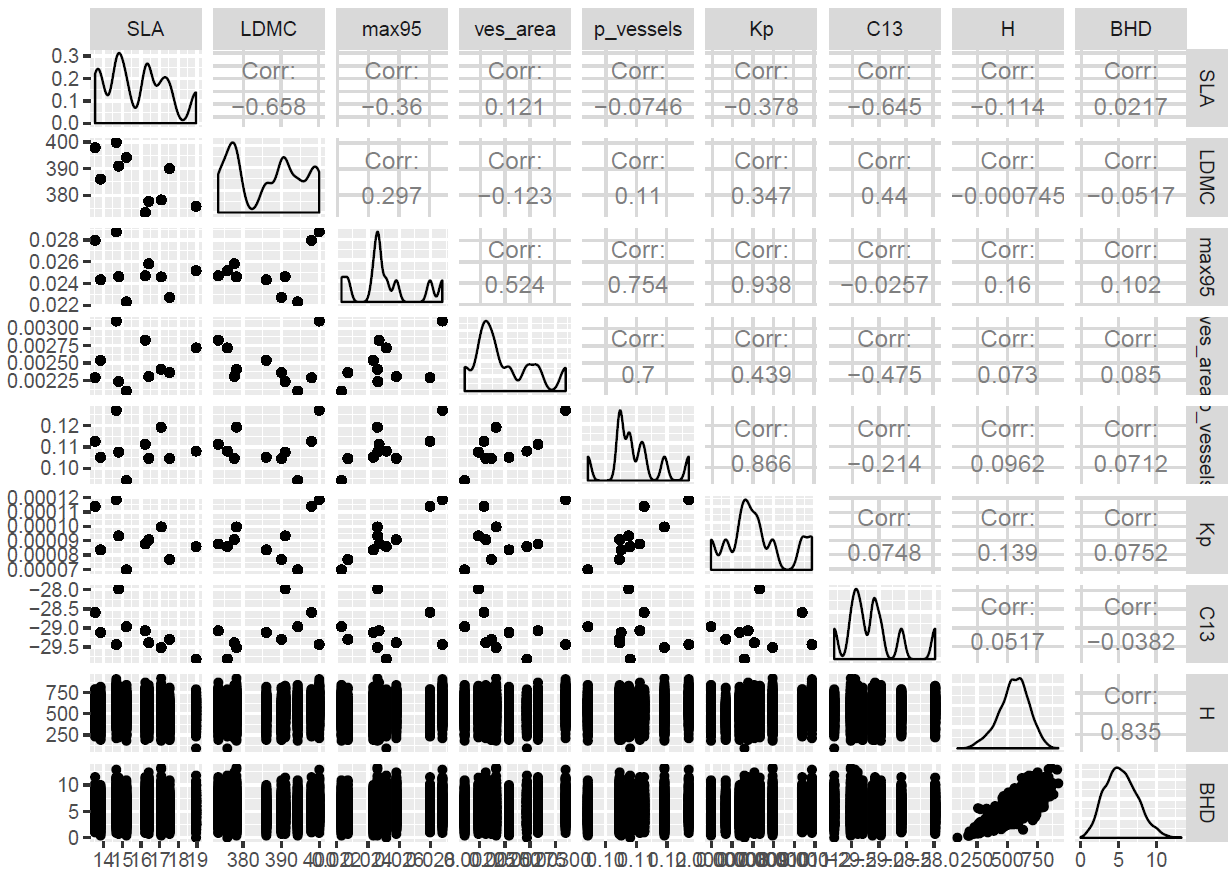


**Fig. S6:** Phenotypic correlations between fitness and functional traits (truncated dataset, functional trait single tree-fitness trait single tree) **-> across all sites**

**Fig. S7:** Phenotypic correlations between fitness and functional traits (truncated dataset, functional trait single tree-fitness trait single tree) **-> dry site**


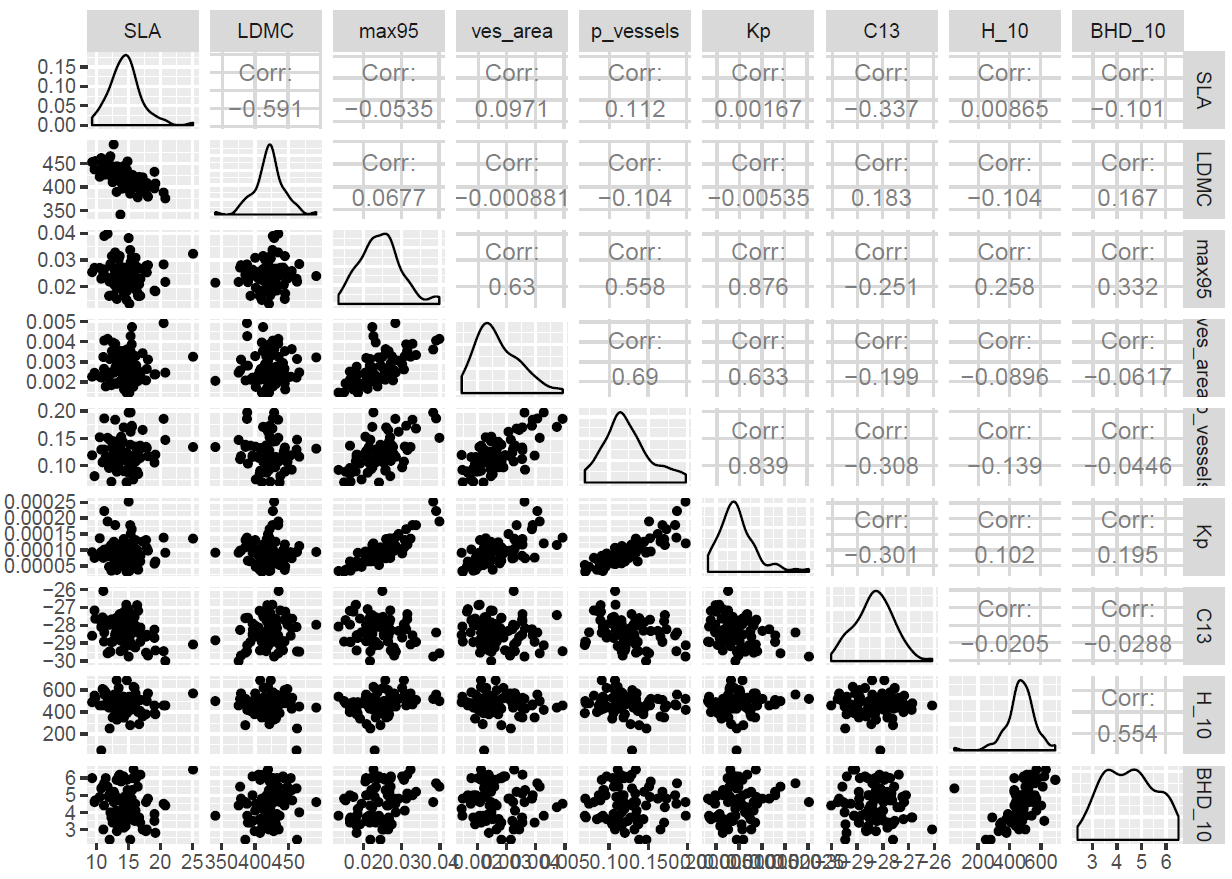


**Fig. S8:** Phenotypic correlations between fitness and functional traits (truncated dataset, functional trait single tree-fitness trait single tree) **-> intermed. site**


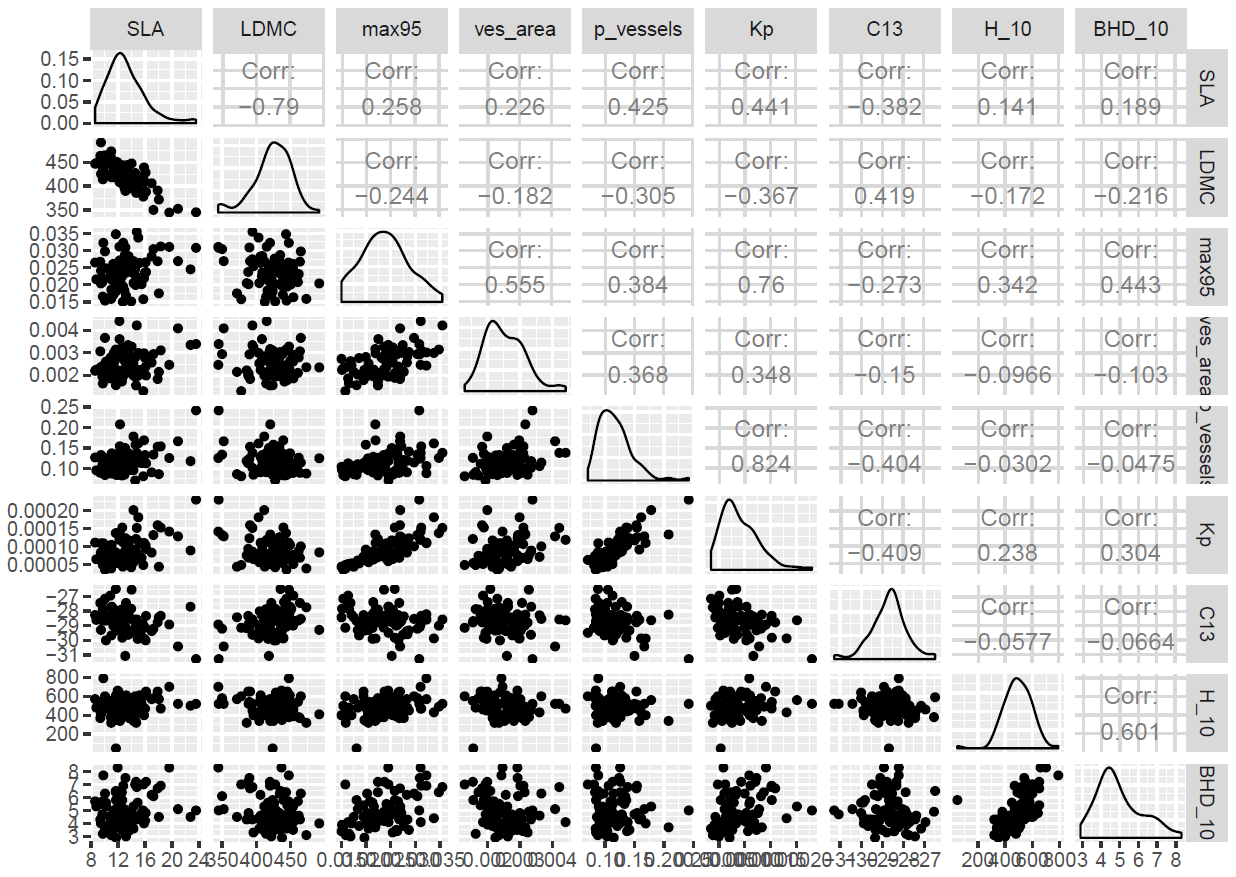


**Fig. S9:** Phenotypic correlations between fitness and functional traits (truncated dataset, functional trait single tree-fitness trait single tree) **-> fresh site**


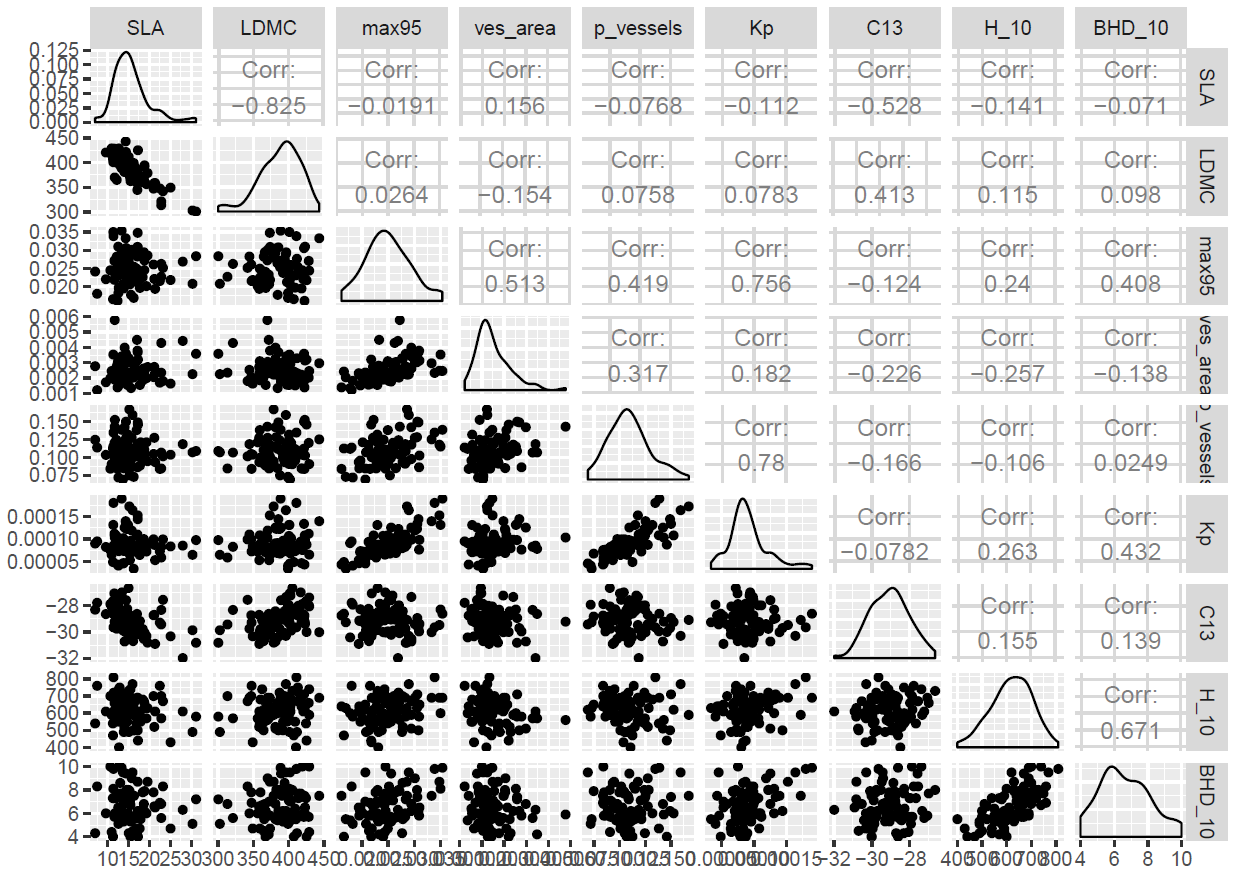

Supplement: Supplementary file 2 — Figs S2–S9 [file EVA-13-2422-s002.doc]
