# Supplementary material for: Assessing adaptive and plastic responses in growth and functional traits in a 10‐year‐old common garden experiment with pedunculate oak (Quercus robur L.) suggests that directional selection can drive climatic adaptation
Source: Evol Appl. 2020 Jun 18;13(9):2422–38. doi: 10.1111/eva.13034 (PMC7513705; doi:10.1111/eva.13034)
Supplement: Supplementary file 3 — Table S1 [file EVA-13-2422-s003.doc]

**Tab. S1:** Climatic variables that were used for assigning provenances to climatic clusters

DD=growing degree days

eFFP=ending date of frost free period

EMT=estimated extreme minimum temperature

FFP=frost free period

MAP/MAT=mean annual temperature/precipitation

MCMT=mean coldest month temperature

MSP=mean summer precipitation

MWMT=mean warmest month temperature

NFFD=number of frost free days
